# Advancing Liquid Biopsy: First Clinical Demonstration of Bio-Ferrography for Isolation and Microscopic Characterization of EGFR-Positive Circulating Tumor Cells in Metastatic Cancer

**DOI:** 10.3390/cancers18142262

**Published:** 2026-07-15

**Authors:** Ofer Levi, Alexander Shtabsky, Baruch Tal, Assaf Shapira, Shiran Shapira, Itai Benhar, Nadir Arber, Noam Eliaz

**Affiliations:** 1 Department of Materials Science and Engineering, Tel Aviv University, Tel Aviv 6997801, Israel; ofemi6674@gmail.com (O.L.);; 2 Pathology Institute, Tel-Aviv Sourasky Medical Center, Tel Aviv 6423906, Israel; 3 The Sackler School of Medicine, Tel Aviv University, Tel Aviv 6997801, Israelnarber@tauex.tau.ac.il (N.A.); 4 The Shmunis School of Biomedicine and Cancer Research, Tel Aviv University, Tel Aviv 6997801, Israelbenhar@tauex.tau.ac.il (I.B.); 5 Integrated Cancer Prevention Center, Tel-Aviv Sourasky Medical Center, Tel Aviv 6423906, Israel

**Keywords:** bio-ferrography, circulating tumor cells (CTCs), colorectal cancer (CRC), epidermal growth factor receptor (EGFR), immunomagnetic separation, liquid biopsy

## Abstract

**Background**: Colorectal cancer (CRC), a leading cause of cancer-related mortality worldwide, necessitates improved non-invasive diagnostic and monitoring tools. Circulating tumor cells (CTCs), as intact cellular biomarkers in liquid biopsies, offer valuable morphological and genetic information and hold significant clinical potential for early detection, prognosis, therapy monitoring, and drug development. Bio-ferrography is a non-invasive immunomagnetic separation technique that isolates magnetically labeled entities from fluid samples onto a glass substrate via a focused external magnetic field. **Methods**: This study employs, for the first time, bio-ferrography for isolation, counting, and microscopic characterization of circulating tumor cells (CTCs) expressing the human epidermal growth factor receptor (EGFR) from blood biopsies taken from patients in the hospital. Magnetic beads conjugated with anti-EGFR antibodies were used to selectively capture CTCs from peripheral blood samples of patients with metastatic CRC and other epithelial malignancies. The method enabled both enumeration and microscopic characterization of isolated cells. **Results**: Preliminary clinical results demonstrate that bio-ferrography achieves a sensitivity of 90% in stage-IV patients and exhibits higher true positive detection rates compared to conventional tumor biomarkers, including carbohydrate antigen 19-9 (CA 19-9) and carcinoembryonic antigen (CEA). **Conclusions**: These findings highlight the potential of bio-ferrography as a robust platform for CTC isolation and analysis.

## Simple Summary

Circulating tumor cells are cancer cells that break away from a tumor and travel through the bloodstream, where they may contribute to the spread of cancer. Detecting these rare cells through a simple blood sample could provide a less invasive way to monitor disease progression and treatment response than conventional tissue biopsies. In this study, we present the first clinical demonstration of bio-ferrography, a magnetic cell isolation technology designed to capture and preserve circulating tumor cells for microscopic examination. Blood samples from patients with metastatic colorectal and other epithelial cancers were analyzed using antibodies that target a protein commonly found on cancer cells. The method successfully isolated and enabled detailed characterization of suspected tumor cells and cell clusters, achieving high detection rates in advanced-stage disease. These findings suggest that bio-ferrography may become a valuable platform for liquid biopsy applications, supporting future research into cancer diagnosis, monitoring, and personalized treatment strategies.

## 1. Introduction

Colorectal cancer (CRC) is the third most frequently diagnosed cancer in men and the second most frequently diagnosed cancer in women [1]. It is the second foremost cause of cancer-related deceases [2]. Circa 30–50% of CRC patients develop regional recurrence or distant metastasis [1,2]. This advises the presence of prospective metastatic cells, which cannot be detected by currently used diagnostic techniques [1]. The five-year survival rate decreases from 90% if CRC is diagnosed at an early stage to 14% in patients with metastatic disease [3]. CRC has therefore been designated of high priority for research due to its combination of high occurrence and lack of diagnostic techniques that are both effective and non/minimally invasive.

CRC usually begins as an adenomatous polyp, which develops into an advanced adenoma with high-grade dysplasia and then progresses to an invasive cancer. In stage 0, cancer superficially involves the mucosa or has not grown beyond it. Invasive cancers that are confined to the wall of the colon (tumor–node–metastasis stages 1 and 2) are curable, but if untreated, they may spread to regional lymph nodes (stage 3) and then metastasize to distant sites (stage 4) [4]. Epithelial cells are the origin of carcinoma cancer types, including CRC. The epidermal growth factor receptor (EGFR) is overexpressed in epithelial carcinoma cells (~10^6^ receptors per tumor cell in comparison to ~10^5^ receptors on a normal cell) [5].

Global CRC screening guidelines recommend colonoscopy (every 10 years), flexible sigmoidoscopy (every five or 10 years), or guaiac-based fecal occult blood test (gFOBT, every one or two years) for average-risk adults aged 50–75 [6,7,8]. Histological examination of the tumor tissue biopsy is conducted to confirm the colonoscopy diagnosis. Besides being invasive, colonoscopy suffers from too low rates of tested individuals, relatively high cost to the health system, need for full bowel preparation, low risk of bleeding, bowel tears or infection, and overlook of small polyps. gFOBT, on the other hand, suffers from high percentage of false positives (2~10% true positive), requires pre-test diet changes, and might overlook many polyps and some cancers. The three aforementioned tests as well as other tests routinely used for early CRC detection, such as double-contrast barium enema, computed tomography (CT) colonography, and stool (or fecal) DNA test, suffer from some significant drawbacks such as non-specificity, extravagant rate of false positives, and high cost [9]. Therefore, there is nowadays a race for the development of new tests for screening and monitoring of CRC.

A blood biopsy screening test based on circulating biomarkers may replace colonoscopy (and other tests) as a first-step screening tool. This test should identify high-risk individuals, which will be followed up by colonoscopy when abnormalities are detected [10]. Liquid biopsy is a minimally invasive and repeatable method characterized by the isolation of cancer-derived components, such as circulating tumor cells (CTCs), circulating tumor DNA (ctDNA), ribonucleic acid (RNA), and proteins, from peripheral blood or other body fluids, and their enumeration, microscopic, genomic or proteomic analysis [3,11,12,13]. Liquid biopsy is of great advantage to patients weakened by either the cancer disease or chemotherapy [14]. However, some limitations need to be overcome before liquid biopsy can be widely incorporated in the clinic [15].

CTCs are tumor cells that detached from the primary tumor site and are transported via the circulation system [16,17]. They can extravasate and become seeds for the subsequent growth of more tumors (metastases) in distant organs. Peripheral blood CTCs can be detected in patients with precancerous colorectal polyps as well as CRC [3,14,16,18]. Detecting the presence of CTCs in the circulating blood outside the primary tumor can thus serve the purposes of monitoring treatment outcome in patients, metastasis prognosis, and even early detection [18,19,20,21,22,23,24,25,26,27,28,29,30]. CTC counts can be used to monitor patients for recurrences [31], or to monitor the biological response of a tumor to treatment and the evolution of its resistance to therapies [32,33,34]. CTC enumeration and characterization may be important for prognosis [2,34]. Compared with other components of liquid biopsy, such as ctDNA or RNA, CTCs are whole cells with unique morphological information and have distinct advantages for evaluating genomics, transcriptomics, and proteomic signaling [2,34].

Unfortunately, the isolation and characterization of CTCs can be challenging due to their large morphological variability and their rare presence in the blood (1–10 per 10^9^ blood cells, thousandth of them white blood cells, in 1 mL human whole blood, HWB) [16,35,36]. CTCs often express a variety of cell-surface markers that vary from patient to patient, making it hard to develop methods with high sensitivity and specificity combined with broad applicability. Furthermore, after release from tumors, most CTCs have short half-life in the circulation (in ~1–2.4 h, compared to <1.5 h for ctDNA) due to mechanical forces or immune system attack [34,37]. Centrifugation, lysis, fixation, or other blood sample manipulation before analysis might decrease significantly the detectable number of CTCs in the sample or damage them [35,37,38]. Moreover, it is very challenging to differentiate between metastatic and nonmetastatic CTCs [37].

CTC clusters (aggregates) are even rarer in the peripheral blood of cancer patients, comprising only 2–5% of the detected CTCs [2]. Compared to individual CTCs, CTC clusters have enhanced metastatic potential, and their presence is closely related to shorter progression-free survival and overall survival in various metastatic cancers [39,40]. Unfortunately, most current CTC detection methods do not allow counting and characterizing CTC clusters [2].

A variety of technologies exist for the isolation of CTCs from patients’ blood samples [16,34,37,38,41]. Immunomagnetic separation (IMS) techniques depend on the affinity between capture ligands and surface receptors of CTCs combined with magnetic trapping [42]. An ideal technology should be able to detect a small number of antigen-positive cells from a background of HWB components. To date, several IMS-based technologies with high recovery rates have been reported. Yet, the only validated method for CTC detection that has been approved by the US FDA is CellSearch™ (Menarini Silicone Biosystems, Florence, Italy) [2,14,16,32,33,35,37,38,43,44,45]. In an evaluation of a prognosis based on CellSearch™ data it has been concluded that patients with a cutoff of five CTCs per 7.5 mL blood have poor survival rate [16]. The main disadvantages of CellSearch™ are that (1) CTCs lacking epithelial cell adhesion molecule (EpCAM) expression cannot be captured, (2) this closed system gives “numbers only” and does not allow easy access to the captured CTCs for complementary molecular, morphological and other characterizations [33,44], (3) the technique has low sensitivity (~30% for CRC patients), (4) the system has low throughput (one sample per run), (5) both the system and the test are expensive ($225k and $1.25k, respectively), and (6) a skilled operator is needed. The CellMax Life’s CMx^®^ CTC assay platform [14,37] was developed for detection of CTCs in early-stage cancer and harvesting of live cells for analysis via gentle airfoam release, without damaging these cells. Reported measures of this technique include a sensitivity of 80% (8 out of 10), cancer cell line cells (CLCs) recovery rate of 63%, specificity of either 56% (18 out of 32) for cutoff 0 or 80% (4 out of 5) for cutoff 2, long test duration (3 days), and modest throughout (5 samples/run) [14].

Ferrography is a magnetic condition monitoring technique that allows the separation of inherently magnetic or magnetized particles from a liquid onto a glass slide under a strong applied magnetic field [46,47,48,49]. The magnetized particles are captured onto a glass slide called a ferrogram, which following magnetic capture is removed from the ferrograph for further processing, usually by microscopy. Ferrography was initially developed to monitor mechanical erosion of metallic devices. Based on the number, size, shape, surface morphology, and chemical composition of the captured magnetic particles, the level, origin and mechanism of wear can be determined. The bio-ferrograph is a modified version of the conventional analytical ferrograph designed specifically to isolate target biological matter such as cells and tissue fragments from body fluids. The advantages of this technology include its extremely high selectivity and sensitivity, very high recovery rates, quantitative analysis of the captured biological matter while observing it microscopically [50], preservation of the structure and morphology of the isolated particles, small and confined deposition area, applicability to any liquid sample (including HWB), and the ability to capture particles as small as several nanometers [51].

To date, bio-ferrography has been used successfully to track bacteria (e.g., [52,53]), capture rare magnetic minerals embedded in Vespinae comb [54], isolate bone and cartilage tissue fragments from the synovial fluids in human joints for diagnosis of osteoarthritis [55], determine the efficacy of hyaluronic acid injections into human knees [48,56], and isolate both polymeric and metallic wear particles from artificial joints for condition monitoring and failure analysis [47,48,57,58,59].

Bio-ferrography has also been utilized to isolate, count and characterize, both microscopically and mechanically, cancer cells. Fang et al. [60] isolated rare MCF-7 breast carcinoma cells from a background of human peripheral leukocytes. The recovery rate of the MCF-7 cells was 20–60%, and the limit of detection (LoD) was one MCF-7 cell in 10^6^ leukocytes. In a second study [61], CD4 cells were isolated from leukocytes and murine lymphoma cells in human peripheral blood. No recovery rate values were reported for that study.

Levi et al. [62] used bio-ferrography to separate target (positive) A431 human epidermoid carcinoma cell line from NIH 3T3 mouse embryo fibroblast background cells. Magnetic labeling was based on (1) the high expression of EGFR on epidermal CTC surfaces [63,64,65]; (2) the ability of high-affinity EGFR-specific antibodies to bind these cells with high affinity and specificity; and (3) the ability of Anti-IgG magnetic-activated cell sorting (MACS™) MicroBeads to capture the antibody-labeled cells. Comparative sample analysis was done using MACS™ (Miltenyi Biotec, Bergisch-Gladbach, Germany) and EasySep™ (Stemcell Technologies, Vancouver, BC, Canada). Next, Levi et al. [66] employed a design of experiments (DOE) methodology to both minimize the number of experiments and optimize the bio-ferrographic CTC isolation procedure. An exceptional recovery rate of up to 97% was achieved when 2–100 CTCs were suspended in 1 mL HWB. For comparison, CellSearch™ was reported capable of capturing at least two CTCs per 7.5 mL blood sample in only 99 out of 333 (~30%) of metastatic CRC patients [43]. The number of captured cells versus input number of cells plot showed a linear dependence, with a slope of 1.006 and *R*^2^ = 0.994 [66]. The aforementioned results highlight the attributes and strengths of bio-ferrography for the isolation of CTCs from HWB and position it as one of the most promising technologies for the detection of CTCs. Svetlizky et al. [67] later used atomic force microscopy (AFM) force spectroscopy to study the mechanical rigidity of individual A431 cells that had been magnetically isolated on a slide by means of bio-ferrography. While the magnetic isolation step itself did not change the elasticity of the captured cells, different steps in the pre-isolation sample preparation steps did. Recently, Eliaz et al. [50] invented a bench-top bio-ferrograph-based high-throughput system that contains 100 flow channels (five channels for each blood sample), allowing the analysis of ca. 20 patients simultaneously. System design was based on the aforementioned optimized procedure for bio-ferrographic isolation of CTCs from HWB.

The exceptionally high recovery rate of A431 cells achieved by bio-ferrography in a lab setting triggered testing of blood biopsies from the clinic in order to further validate the potential of bio-ferrography as a tool for cancer diagnosis and estimation of prognosis. To the best of our knowledge, the applicability of bio-ferrography to cancer monitoring (or screening) of patients has never been demonstrated before. The objective of this feasibility study was thus to employ, for the first time, bio-ferrography for isolation, counting, and microscopic characterization of CTCs from blood biopsies taken from patients in the hospital. Cytological analysis of the captured CTCs was conducted. This preliminary study paves the way for further research and development, which may open the gate in the future for routine use of bio-ferrography-based methods in the clinics.

## 2. Materials and Methods

### 2.1. Blood Samples from Cancer Patients

A total of 54 blood samples from 48 cancer patients were provided by the Integrated Cancer Prevention Center (ICPC) at Tel-Aviv Sourasky Medical Center (see Table 1). After completion of the experimental work, the medical files of 43 patients (49 blood samples) were fully retrieved; these are listed in Table 1. In this table, gastrointestinal (GI) cancers gather colorectal, colon, rectal and stomach cancers together. From Table 1 it is evident that 4 patients turned out to be associated with non-CRCs (pancreas, duodenum, esophageal, or lung).

**Figure 1:**
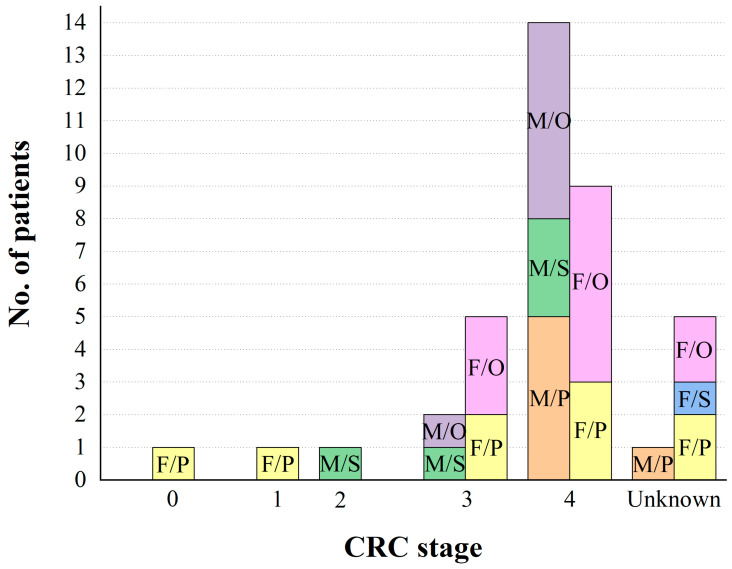
CRC stage distribution among male and female patients. M—male, F—female; P—preliminary phase, S—screening phase, and O—optimized and validated phase in the DOE (these phases are described in Ref. [66]). A preliminary phase was employed for minimizing the number of factors. The screening phase aimed to evaluate the effect of eight experimental factors on the recovery rate. The optimization phase focused on the three critical factors identified by the screening phase.

Figure 1 shows the stage distribution for CRC patients (either as primary or secondary tumor, based on the medical files), divided according to gender. Evidently, most samples were drawn from patients in an advanced stage of disease (stages 4 and 3).

### 2.2. Blood Samples from Healthy Donors

HWB samples from three healthy donors were pre-tested and supplied by the MDA Israel Blood Services. These HWB samples were used as negative controls that were run on one of the five channels on some ferrograms (1 mL blood per channel).

### 2.3. A431 Cell Line Positive Controls

Cells of the A431 epidermoid carcinoma cell line were used as positive controls for EGFR-overexpressing target cells. They were processed essentially as described in Refs. [62,66]. Briefly, the A431 cells were maintained in Dulbecco’s Modified Eagle Medium (DMEM) supplemented with 10% fetal calf serum (FCS), 2 mM L-glutamine, 100 U/mL penicillin, 100 μg/mL streptomycin, and 12.5 U/mL nystatin (Biological Industries, Israel) in a humidified 5% CO_2_ incubator at 37 °C. The A431 cells were transfected to stably express a red fluorescent protein (mCherry). The A431 target cells were fixed with 4% formaldehyde prior each isolation procedure. The 1 mL patient blood samples were spiked with 100 A431 cells each and were run on one of the channels in some of the ferrograms. This was used as a positive control, recovery rate control, and as a reference for target cells.

### 2.4. Bio-Ferrographic IMS

**Figure 2:**
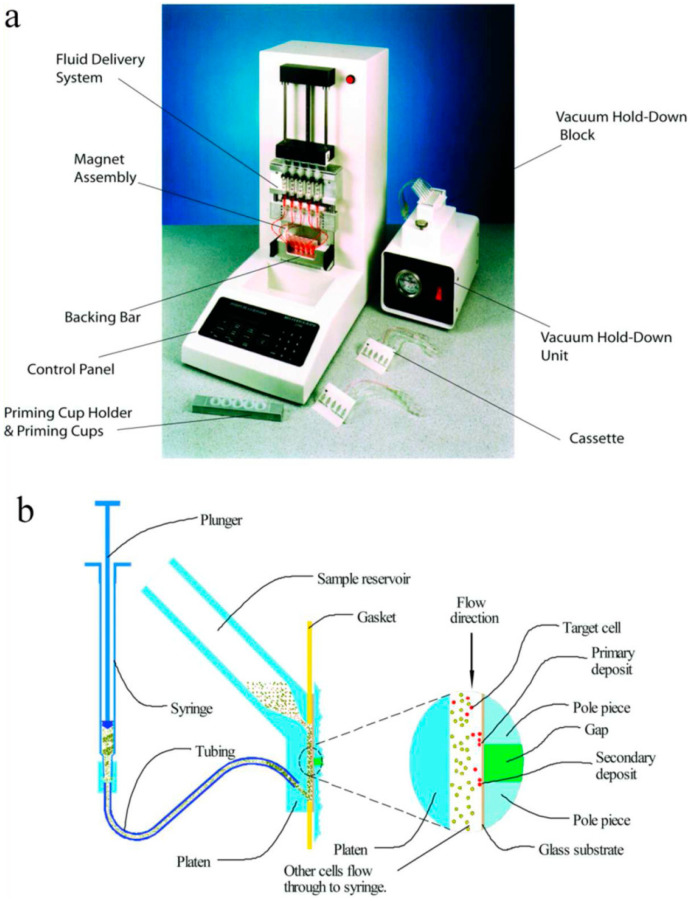
(**a**) The Bio-Ferrograph 2100 (Guilfoyle, Inc.) and its components. (**b**) The fluid sample flow and deposition scheme of captured particles on the slide [62,66].

The CTCs isolation procedure comprised an immunomagnetic labeling stage and a magnetic bio-ferrographic isolation stage:

(1) Ab EGFR (R-1) mouse anti-human IgG antibody (Santa Cruz Biotechnology, Inc., Santa Cruz, CA, USA, catalog No. sc-101, 0.2 mg/mL) was mixed with Miltenyi MicroBeads conjugated to monoclonal rat anti-mouse IgG antibodies (Miltenyi Biotec, Inc., Auburn, CA, USA) and incubated at 7 °C for 20 min (this will be referred to hereafter as the cocktail suspension). Each patient’s blood sample was separated and run on three channels on the ferrogram. The remaining two channels were used for negative and positive cell controls. The positive control sample was spiked with 100 positive control cells (A431). For this purpose, at least three drops were first drawn onto a glass slide in order to verify the inlet amount of 100 spiking target cells. The target cells per each drop were counted and averaged. The average value (namely, “the inlet amount of target cells”) was used for the recovery rate calculation. The blood samples were washed (without any red blood cell lysis process) and were incubated with the cocktail suspension at 20 °C using an orbital shaker and mild shaking.
(2) For cell isolation from blood, a Bio-Ferrograph 2100 (Guilfoyle, Inc., Belmont, MA, USA) was used. A photograph of the instrument, together with a schematic representation of the deposition pattern of captured particles on the slide, is shown in Figure 2. In order to minimize contamination of the samples by the surroundings, the Bio-Ferrograph was placed in a biological safety cabinet (ADS Laminar’s Optimale 12). All samples were washed prior to bio-ferrographic separation and filled with phosphate-buffered solution (PBS) to a fixed volume of 0.5 mL. During the bio-ferrographic separation process, the capture cell and reservoir were filled with 0.5 mL PBS at a flow rate of 0.1 mL/min. This was followed by inserting the sample with the target cells into the reservoir and isolating the target cells on the capture band at a pre-defined flow rate. Finally, the chambers were washed with PBS at the same flow rate.

The blood samples were isolated following one of three types of isolation procedures (preliminary, screening, and optimized and validated [66]; see Figure 1 and Table 2), each type having its characteristic recovery rate. The entire blood sample was separated into as many as three replicates. Each patient’s HWB sample was analyzed on one ferrogram. The cells on the ferrograms were immediately fixed in 4% paraformaldehyde (PFA), which was found to yield better results than either immediate fixation in ethanol or air-drying followed by fixation in methanol.

### 2.5. CTC Characterization

The ferrogram with the isolated cells was separated from the bio-ferrograph and examined under an inverted fluorescence microscope (Olympus IX71, Tokyo, Japan). The recovery rate (R.R.) was calculated according to Equation (1):

(1) $$\mathrm{Recovery}\;\mathrm{rate}\;(\%)=\frac{\#\;\mathrm{of}\;\mathrm{cells}\;\mathrm{observed}\;\mathrm{within}\;\mathrm{the}\;\mathrm{capture}\;\mathrm{field}}{\#\;\mathrm{of}\;\mathrm{cells}\;\mathrm{counted}\;\mathrm{at}\;\mathrm{the}\;\mathrm{inlet}\;\mathrm{of}\;\mathrm{the}\;\mathrm{process}}\times 100$$

The identification of the captured positive control cells (A431) was straightforward because they were transfected to stably express a red (mCherry) fluorescent protein prior to the bio-ferrographic isolation. Isolation of CTCs from patient’s HWB samples, on the other hand, required a special procedure in order to identify unambiguously the CTCs within the capture band on the ferrogram. To this aim, several approaches were tested. Eventually, the combination of fixation with 4% PFA in the final stage of isolation, hematoxylin and eosin (H&E) staining, and morphological (cytological) analysis was found to be most suitable for CTCs identification and characterization.

The ferrograms were analyzed by an expert pathologist (A.S.). Seven analysis categories of cell types were defined: blood cells only (0); cells of undetermined significance, apparently blood cells (1A); cells of undetermined significance (1B); atypical cells of unknown significance (2); atypical cells suspected for malignancy/malignant (3); failed due to poor preservation conditions (F); false positive, related to the control channels with healthy blood (F.P.).

Since CTC identification was based on morphological analysis, the main cytological characteristics for consideration were first defined, as follows:

• Cell size (diameter)—the CTC origin is from an epithelial tumor tissue. These cells are typically enlarged and have approximately three times larger diameter in comparison with normal blood cells (erythrocytes (or RBCs), granulocytes, lymphocytes, etc.) [68].
• Nucleus-to-cytoplasm ratio—epithelial CTCs have enlarged nuclei that leave a very small space for the cytoplasm. In comparison to healthy epithelial cells and nucleated blood cells, epithelial CTCs typically have a large nucleus-to-cytoplasm ratio [68].
• Indentations or projections of nuclear membrane—the nuclear membrane of epithelial CTCs loses its roundness, and some indentations and projections might appear [69].
• Hyperchromatic nucleus—the CTC’s nucleus has a dark and shiny hue in comparison to other nucleated blood cells.
• Aggregation—due to the adhesive properties of the epithelial cell, CTCs’ disconnection from their origin tumor to the blood circulation might appear as aggregates.
• Mitosis—CTCs that separate from their original site might be in a process of division into two or more daughter cells (cell division).

**Table 2:** Bio-ferrographic analysis and tumor markers of the patients from Table 1.

Patient #	Isolation Phase	R.R. Positive Control	Patient’s Bio-Ferrographic Classification ^1,5^	Channel 1 ^1,2,5^	Channel 2 ^1,2,5^	Channel 3 ^1,2,5^	Channel 4 ^1,2,5^	Channel 5 ^1,2,5^	CA 19-9 (0–37 U/mL) ^3^	CEA (0–5 ng/mL) ^3,4^	AFP (0–20 ng/mL) ^3^	Other Markers
1	P	-	1A	1A (1)	0	0	0	1A (1)	No			
2	P	-	1A	0	0	0	1A (>1)	0				
3	P	-	0	0	0	0	0	0				
4	S	-	1B	0	1B (>1)		1B (1)	F	64	6		
5	O	81%	F	H	C_R.R.	F	F	F				
6	O	97%	0	H	C_R.R.	0	0	0	8	2		
7	O	-	F	C	F	F	F	F				
8-1	O	-	3	C	2 (>1)	C	0	3 (1)	11–13	1.5–3		
8-2	O	69%	1B	H	C_R.R.	1B (>1)	1B (>1)	1B (>1)	11–13	1.5–3		
9-1	P	-	1B	0	1B (>1)	1B (>1)	1B (>1)	1B (>1)	9	2.5–4		
9-2	P	-	1A	1A (>1)	1A (>1)	0	1A (>1)	1A (>1)	9	2.5–4		
10	O	-	F	F	F	C	F	F				
11	O	-	F	F	F	F	F	F				
12	P	-	0	0	0	0	0	0	9	0.8		
13	O	-	0	0	0	C	0	0	33	<1.3		
14	P	-	0	0	0	0	0	0	8–12	2–10		
15	P	-	0	0	0	0	0	0	89	2.5		
16	O	93%	3	H	0	C_R.R.	3 (1)	0	28	<1.3		
17	O	97%	3	H	2 (1)	C_R.R.	3 (3)	0	4–138	4–15		
18	S	-	F	F	F	F	F	F				
19	O	84%	3	H	3 (>10)	C_R.R.	1A (>1)	1A (>1)	16–49	7–41		
20	O	57%	2	H	C_R.R.	2 (3)	2 (>1)	2 (>1)	14	<1.3		
21	P	-	1B	1B (>1)	1A (>1)	1A (>1)	0	0	No			
22-1	P	-	1B	0	0	0	0	1B (1)	9–13	3.9–4.8		
22-2	P	-	2	0	0	0	2 (1)	0	9–13	3.9–4.8		
22-3	P	-	1B	F	F	1B (>1)	0	0	9–13	3.9–4.8		
23	O	-	3	1B	2 (1)	2 (2)	3 (1)	0	127–3878	59–1606		CA125: 105.6; CA15-3: 6.4
24	O	-	3	C	3 (>1)	3 (>3)	1B (>1)	F	7	2.4		
25-1	S	-	0	0	0	0	0	0	4272	665	0.75	
25-2	P	-	F	F	F	F	F	F	4272	665	0.75	
26	P	-	3	3 (2)	0	0	0	0	12	1.4		
27	S	-	1A	0	0	0	1A (1)	0				
28	O	-	3	3 (5)	3 (5)	3 (5)	C	3 (5)	109	2.4		
29	P	-	2	2 (4)	2 (7)	2 (2)	1B (>1)	1A (4)	288	26		
30	S	-	0	0	0	0	0	0				
31	P	-	1B	1A (1)	1B (1)	0	0	0				
32	O	-	3	0	3 (1)	C	F	F	606	1305	1.83	CA15-3: 30.6
33	O	-	F	C	F	F	C	C				
34	S	-	2	0	0	2 (2)	1A (>1)	1A (>1)	1504	302	1.63	CK & CK7: positive; HER2: negative
35	P	-	0	0	0	0	0	0	192	380	2.4	CA125: 470, CA15-3:
36	O	-	3	3 (3)	1B (2)	1B (>1)	C	C	298	34		
37-1	P	-	F	F	F	F	F	F				
37-2	P	-	2	2 (>1)	2 (>1)	0	0	0	9	2		
38	S	-	1A	0	0	0	1A (2)	0	10	14		CA125: 22.7
39	O	-	F	F	F	F	F	C				
40	O	93%	3	H	C_R.R.	3 (1)	3 (2)	3 (3)	5	1.3		
41	S	-	0	0	0	0	0	0	0	<1.3		
42	O	94%	0	H	0	C_R.R.	0	0	>13,200	>13.5		
43	O	-	1B	0	1B (>1)	1B (>1)	C	C	1490	<1.3		

^1^ The patient’s bio-ferrographic classification and the classification of each channel on the ferrogram are in accordance with the seven analysis. Categories based on cell morphology, as listed in the text. ^2^ The numbers in parentheses are the number of cells of the specific category that were identified visually within that channel on the ferrogram. ^3^ The numbers in parentheses below the tumor markers are the normal levels for adults. ^4^ The normal level for a smoker adult. The normal level for a non-smoker adult is 0–2.5 ng/mL [70]. ^5^ H—healthy donor, C—positive control (A431), C_R.R.—positive control analyzed for recovery rate, F—failed sample.

### 2.6. Patients’ Medical Files

Once all experimental work was completed, the patients’ medical files were examined for complimentary data, such as patient’s condition after ca. one year (live or dead); medical treatments (chemotherapy, radiation, surgery, etc.), if given; a sign for EGFR positive response and the values of the tumor markers that were used to monitor the progression of disease (CA 19-9, CEA, alpha-fetoprotein (AFP), cancer antigen 125 (CA 125), etc.).

## 3. Results

Each of the patient’s three channels and the negative control channels on the ferrogram were classified according to the seven aforementioned analysis categories. Each patient was then associated with the most severe rank in any of the three channels. Table 2 summarizes the observations in all channels of the ferrograms of all patients, the classification of each patient based on the bio-ferrographic analysis, the related phase of the isolation protocol, the recovery rate (R.R.) measured for the A431 cells (positive controls) in one of the channels on the ferrogram, and the sampling of negative controls (healthy donors, H). The values of tumor markers are included for comparison, whenever they were indicated in the medical file of the patient. The patient numbers match those in Table 1.

Sensitivity: From Table 1 and Table 2 is it evident that the one stage-0 CRC patient (patient #1) was classified according to the bio-ferrographic analysis at category 1A. The stage-1 CRC patient (#21) and the stage-2 CRC patient (#4) were both classified as 1B. It should be noted, however, that only the preliminary (P) and screening (S) phases, respectively, were employed in these cases, which could lead to loss of some valuable cells. Out of the seven stage-3 CRC patients (#5–8, 22, 37, 38, only the first four of which were isolated by the optimized and validated phase), two were disqualified due to poor preservation condition, one was classified as 0, one was classified as 1A, two were classified as 2, and one was classified as 3. Out of the 23 stage-4 CRC patients (#9–19, 23–29, 32–36), four were disqualified, six were classified as 0, one was classified as 1A and one as 1B, two as 2, and nine were classified as 3. However, when distinguishing between DOE phases O, S, and P, we get 8/9, 0/2, and 1/8 classification 3, respectively. Out of the six CRC patients with an unknown stage (#2, 3, 20, 30, 31, 39), one was disqualified while the rest were classified as 0, 0, 1A, 1B and 2.

**Figure 3:**
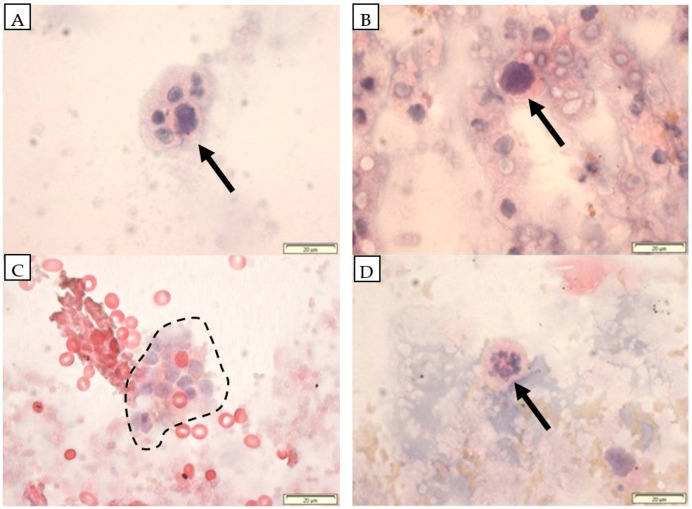
Representative cell morphologies on the ferrogram after bio-ferrographic IMS. (**A**) CTC (arrow) surrounded by mononuclear leukocytes. CTC demonstrates large cell size, increased nucleus–cytoplasmic ratio and large hyperchromatic nucleus with projections and indentations of the nuclear membrane. (**B**) Hyperchromatic nucleus. Target cell (arrow) among blood cells. Note size differences and small nuclear membrane indentations. (**C**) Aggregation. Suspected CTC aggregates (surrounded by dashed line) among erythrocytes. (**D**) Mitosis. Mitotic division in the CTC (arrow). The blurred, fuzzy cytoplasmic contour indicates poor preservation condition. Scale bar: 20 μm.

Representative morphological (microscopic) characteristics of cells after isolation by bio-ferrography are demonstrated in Figure 3. As explained in the Introduction, CTC clusters (aggregates) as in Figure 3C have enhanced metastatic potential [39,40], yet most current CTC detection methods do not allow counting and characterizing them [2].

Negative controls: HWB samples from three healthy donors, which are not included in Table 1 and Table 2, were not spiked with any cells (such as A431). They were run on the bio-ferrograph, following the same, full procedure used for cancer patients. Hence, they were used as negative controls. In none of these three ferrograms were suspicious cancer cells found (i.e., specificity of 100%, or 3 out of 3, for cutoff zero). The high specificity of the bio-ferrographic analysis is further supported by the H notation in some cells in Table 2. Out of nine ferrograms with H channel, suspicious cells were observed only in one case (patient #20). Patients #42 and #43 may be regarded as another type of negative controls. Both suffered from stage-4 non-epithelial tumors (pancreas and pancreas and duodenum, respectively), and were classified by bio-ferrography as 0 and 1B, respectively (no suspected malignant cells were observed on their ferrograms). This is what one could expect of IMS which used EGFR biomarker, which is overexpressed on the surface of epithelial carcinoma cells. It should be emphasized that the classification of non-epithelial tumor patients as category 0 or 1B provides highly compelling biological evidence for the specificity of EGFR capture.

Positive controls: A 1 mL patient blood sample was spiked with 100 A431 cells, which were then recovered within a channel on the ferrogram simultaneously with the run of four other blood samples (see notations C and C_R.R. in Table 2). In all cases, A431 cells were indeed identified morphologically on the ferrogram under the microscope. The ferrograms of 7 out of 39 CRC patients included a channel for R.R. measurements, employing the optimized phase (O) as was previously determined by DOE [66]. Values between 57% and as high as 97% were measured. Furthermore, R.R. values of 93% and 94% were measured when using blood samples of two non-CRC patients (with esophageal and pancreas cancers, respectively), when also employing the optimized phase in the DOE.

**Figure 4:**
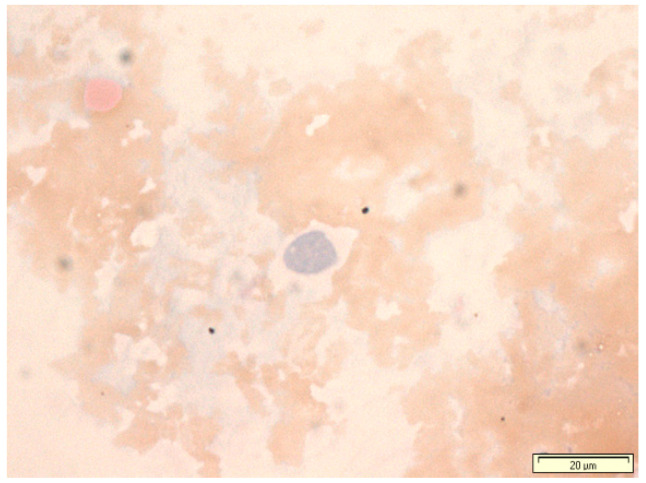
Poor preservation of a cell. No cytoplasm membrane can be noticed.

Comparing Table 2 to Table 1, it is apparent that nine ferrograms of CRC patients were excluded from further analysis due to poor microscopic condition of the captured cells (see Figure 4), which precluded a definite pathological decision. We believe that this poor condition resulted from poor preservation of the blood samples between their drawing at the hospital and the bio-ferrographic isolation procedure that was carried out at a different location (the University campus), either during storage or during handling.

Table 3 summarizes the main measures in this study. The optimized (and validated) procedure (O) was found helpful in increasing the sensitivity, as was previously concluded from DOE [66], up to 90% in stage-4 epithelial cancers, at the highest level of confidence (category 3). Bio-ferrography was found to have significantly higher success rates in detecting stages 3 and 4 cancers compared to the tumor markers CA 19-9 and CEA (as well as others such as AFP), which are commonly used for monitoring or recurrence of CRC patients [71,72,73]. It can be concluded from Table 3 that bio-ferrography may offer both high sensitivity and high specificity in monitoring colorectal and other epithelial cancers.

## 4. Discussion

In our past publications on the use of bio-ferrography for cancer diagnosis we first developed the protocol for the magnetization and isolation of A431 model cells from either PBS or healthy HWB using anti-EGFR antibodies conjugated to magnetic beads [62]. We then implemented DOE to increase the recovery rate, from 46% in the initial (preliminary, P) stage to 81% after the screening phase (S), to 95% after the optimization phase (O), and to 97% after the validation stage [66]. The P, S, and O phases were also compared in the current study. The exceptionally high recovery rate after the validation phase was achieved when spiking 1–100 A431 cells in 1 mL WHB. The typical sample volume for CellSearch™, for comparison, is larger (7.5 mL), while the LoD of EasySep™ and MACS™ (1 × 10^5^) does not allow isolation and characterization of a few CTCs [62]. In yet another study [67], we studied the effects of different stages in the pre-isolation process as well as the bio-ferrographic isolation itself on the mechanical rigidity of the captured A431 cell on the ferrogram. Recently, we designed, both mechanically and magnetostatically, a novel high-throughput semi-automated instrument with 100 flow channels (compared to five in the Guilfoyle’s bio-ferrograph) [50]. The new instrument is intended to allow analysis of blood samples from 20 patients simultaneously, on-site where the blood samples will be drawn. Here, we report for the first time the results of our clinical feasibility tests.

Here, we succeeded in reproducing the high recovery rate from our past study in five out of nine patients, this time when spiking the A431 cells in patient’s blood and employing the O phase. Ignoring for a moment the small sample size, the O-phase of our bio-ferrographic procedure achieved sensitivity of 90% for stage-4 epithelial cancer patients, much higher than that of the CA 19-9, CEA and AFP tumor markers that were used in the hospital for diagnosis and prognosis of some of the same patients. At least one CTC could be identified unambiguously on the ferrograms of the 9/10 stage-4 patients (microscopically, by a pathologist visual analysis) after running 1 mL only. All positive and negative controls (that included specificity testing at zero cutoff) yielded the expected conclusions, except one healthy blood sample (out of nine H-samples) that revealed some suspicious cells. As mentioned before, CellSearch™, the only FDA-approved CTC detection technology, has proved a sensitivity of only ~30% from 7.5 mL blood samples of metastatic CRC patients [43]. CellSearch™ relies primarily on the capture of EpCAM-positive epithelial CTCs and is therefore constrained by epithelial-to-mesenchymal transition (EMT), tumor heterogeneity, and relatively limited sensitivity in several malignancies. In contrast, our BF platform is designed to detect a broader cancer-associated cellular and immunological signature, potentially extending beyond conventional epithelial CTC identification. Direct head-to-head comparisons with established technologies such as CellSearch™ would further strengthen the translational positioning and clinical validation of the BF platform. Unfortunately, such comparative studies were not feasible in the present work because a CellSearch™ system is not available in Israel [62].

This puts our results in a proper context and indicates the great potential of bio-ferrography in monitoring (and, perhaps, also screening) epithelial cancer patients for simple blood biopsies. The small blood sample volume required and the rapid sampling time could, in the future, enable monitoring of CRC patients during outpatient clinic visits, allowing non-invasive assessment of treatment response or disease progression.

Despite the aforementioned success of bio-ferrography in this study, several shortcomings should be resolved before the technology can be considered seriously for widespread application in the clinic. Many of the analyses were based on only one suspected CTC (or a few). This very small number of captured suspected carcinoma cells calls for further adaptation of the procedure in order to increase the number of captured cells, thus making microscopic analysis easier and more statistically significant. Some of the possible ways to achieve this include: (1) Increasing the the sample volume to 5 mL per tube. (2) Identify a better immunofluorescence, immunohistochemistry, or cytological approach for specific targeted staining with higher signal-to-noise ratio. This may require the use of other antibodies. While H&E staining gave significantly better results in this study than the immunofluorescence approach, an even better approach may be available. (3) Incorporation of artificial intelligence (AI) techniques, including machine learning (ML), may make CTC counting fully automatic, increase the sensitivity and specificity, and facilitate early diagnosis of cancer, although their use in primary care settings is still at an early stage of maturity [74,75,76,77,78,79]. (4) The ability to characterize microscopically, biologically, chemically, and mechanically individual cells captured on the ferrogram while preserving their original shape is an important advantage of bio-ferrography compared to most other IMS techniques. However, the poor preservation of cells that was found during analysis of some patient samples may explain the difficulties in immunofluorescence analysis. Damaged cell cytoplasm could prevent receptors from being conjugated and decrease the sensitivity. Poor sample preservation should be prevented via optimization and standardization of storage, transportation and handling of blood samples. In addition, placement of the bio-ferrograph in the hospital could contribute to this effort. (5) We have used the EGFR biomarker. However, overexpression of EGFR was detected in only 49–82% of CRCs [80,81,82,83]. Other biomarkers (e.g., EpCAM, the cytoskeletal proteins—CK8, CK18, and CK19, epithelial-to-mesenchymal transition (EMT), mucin 1 (MUC1), the carbohydrate antigen 199 (CA19-9), etc. [11,37,38,73,84,85]) may thus turn out to yield higher sensitivity in bio-ferrographic-based IMS. (6) Last but not least, this research should be extended to significantly larger populations of patients, not only with stages 2–4 cancers but even more challenging—stages 0 and 1. It should be emphasized that the aforementioned six future improvement directions are not merely aspirational future goals, but rather prerequisites revealed by this preliminary study that must be addressed before clinical translation can be considered.

One may argue that in times when DNA tests have become very popular and offer exceptionally high sensitivity, there is no longer a need for new CTC isolation technologies. However, an in-depth analysis of the recent literature [2,3,11,12,15,33,34,36,38,84,86,87,88,89] reveals that this is not the case, and that both approaches may be regarded as complementary [2,89]. When a cell dies, it releases circulating free DNA (cfDNA) into the bloodstream. When this phenomenon originates from primary tumors, metastases, or CTCs, it is called ctDNA. The major advantage of ctDNA is that it is more easily detected in plasma or serum than CTCs [2,11,34]. In addition, ctDNA can provide information about the tumor genome without the need to isolate CTCs [34]. ctDNA phylogenetic analysis can depict early stages of cancer evolution and illuminate the therapeutic resistance of tumors to chemotherapy through genetic mutations [33,34,38]. It outperforms CTCs for KRAS mutation detection in both diagnostic sensitivity and specificity [2]. Yet, the utility of ctDNA in the clinics remains controversial due to the following reasons. (1) Elevations of ctDNA might reflect physiologic or pathologic processes that are not disease-specific, e.g., inflammatory disease or tissue trauma, yielding false positives [2]. (2) Low amounts of mutant fragments in the total cfDNA sample limit the detection success and give rise to false negative results [15]. (3) Background noise results from non-neoplastic age-dependent clonal expansion. (4) Due to the DNAse activity, there is a fairly short time limit between blood collection and the processing of the blood for ctDNA analysis [36]. (5) Plasma ctDNA concentrations in cancer patients range from a few ng/mL to several thousand ng/mL, which overlaps the range for healthy individuals [3,11]. (6) Each ctDNA assay has its own specification, making the comparison between results obtained from different liquid biopsy platforms not easy [3]. (7) While CTCs can be cultured (e.g., for drug development and personal treatment compliance), ctDNA cannot be cultured at all [34]. (8) CTCs can also be used for DNA analyses and to provide information gathered from their protein and RNA content; some of this information is unobtainable from ctDNA [2,34,86]. (9) cfDNA alone is not appropriate for evaluation of tumor stage [86], for example differentiation between stages 1/2 and 3/4 during adjuvant therapy. In the bottom line, while CTCs reflect more of the metastases-initiating cells, ctDNA signifies more the tumor burden [2]. Of course, one has to note also the key limitations of CTCs isolation and analysis: (1) rare presence in blood, (2) morphological variation, (3) they often express a variety of markers, (4) they die in the circulation within 1–2.5 h, and (5) it is necessary to differentiate between metastatic CTCs and nonmetastatic CTCs.

In conclusion, the applicability of bio-ferrography combined with the epidermal growth factor receptor (EGFR) biomarker for isolation from blood biopsies taken from patients in the hospital, counting and microscopic characterization of CTCs for monitoring patients with colorectal cancer (CRC) and some other epithelial cancers was demonstrated. Preliminary results showed sensitivity of 90% (optimized and validated procedure in the design of experiments, DOE, and the highest level of confidence) for stage-4 patients, along with exceptional specificity, using 1 mL blood only. Bio-ferrography was found to have significantly higher success rates in detecting stages 3 and 4 cancers compared to the tumor markers CA 19-9 and CEA (as well as others such as AFP). This study paves the way for further research and development, which may open the gate in the future for routine use of liquid biopsy and bio-ferrography-based instrumentation in the clinics for cancer monitoring.

## 5. Conclusions

This study demonstrates the applicability of bio-ferrography combined with the epidermal growth factor receptor (EGFR) biomarker for isolating circulating tumor cells (CTCs) from blood biopsies of hospitalized patients. This approach enables efficient enumeration and microscopic characterization of CTCs for monitoring colorectal cancer (CRC) and other epithelial malignancies. Preliminary findings indicate a sensitivity of 90% for stage-4 patients—based on an optimized and validated design of experiments (DOE) protocol—alongside exceptional specificity, using only 1 mL of blood. Notably, bio-ferrography exhibited significantly higher detection success rates for stage-3 and -4 cancers compared to conventional tumor markers such as CA 19-9, CEA, and AFP.

## Figures and Tables

**Table 1:** Information on the analyzed cancer patients.

Patient #	Age	Gender ^1^	Cancer Type ^2^	Stage	Surgery Before Sampling	Treated With	Patient Status ^3^
1	82	F	NF1 ^4^	0	No	-	L
2	70	F	NF2 ^4^	Unknown	No	-	L
3	64	M	NF3 ^4^	Unknown	No	-	L
4	67	M	GI	2	Yes	Chemotherapy	L
5	53	F	GI	3	Yes	Adjoventi, FOLFOX ^5^, Erbitux	L
6	58	F	GI	3	Yes	FOLFOX ^5^, Erbitux	L
7	62	F	GI	3	Yes	FOLFOX ^5^	L
8-1	66	M	GI	3	Yes	Chemotherapy	L
8-2							
9-1	84	M	GI	4	Yes	FOLFOX ^5^, Avastin^®^, 5-FU ^5^, LCV ^5^, Biological Treatment	L
9-2							
10	80	F	GI	4	Unknown	5-FU ^5^	D
11	77	F	GI	4	No	-	D
12	70	F	GI	4	Yes	FOLFOX ^5^, Avastin^®^	L
13	67	F	GI	4	Yes	FOLFOX ^5^, Avastin^®^	L
14	64	F	GI	4	No	FOLFOX ^5^, Avastin^®^; later: Sirtex (with no Avastin^®^)	L
15	85	M	GI	4	No	5-FU ^5^, LCV ^5^, Avastin^®^	D
16	76	M	GI	4	Yes	FOLFOX ^5^, Avastin^®^	L
17	51	M	GI	4	Yes	FOLFOX ^5^, Avastin^®^, anti-VEGF, Erbitux, Folfiri	L
18	48	M	GI	4	Yes	Clexane	L
19	40	M	GI	4	Unknown	Unknown	L
20	75	F	GI	Unknown	Yes	5-FU ^5^, Leucovorin	L
21	71	F	GI	1	No	-	L
22-1	60	F	GI	3	No	Chemotherapy	L
22-2							
22-3							
23	70	F	GI	4	Yes	FOLFOX ^5^	L
24	69	F	GI	4	Yes	Unknown	L
25-1	88	M	GI	4	No	-	D
25-2							
26	73	M	GI	4	Yes	Unknown	L
27	58	M	GI	4	Unknown	Unknown	Unknown
28	54	M	GI	4	Yes	-	D
29	64	M	GI	4	Yes	-	L
30	59	F	GI	Unknown	No	-	L
31	51	F	GI	Unknown			L
32	70	M	GI	4	No	5-FU ^5^, Cisplatin, Radiation Treatment	D
33	67	M	GI	4	Yes	Chemotherapy, Bisphosphonates	L
34	64	M	GI	4	No	-	D
35	26	F	GI	4	No	-	L
36	67	F	GI	4	No	FOLFOX ^5^, Avastin^®^; later: 5-FU ^5^, Leukovorin	D
37-1	69	F	GI	3	Yes	FOLFOX ^5^, 5-FU ^5^, Leucovorin, Chemotherapy	L
37-2							
38	38	M	GI	3	Yes	FOLFOX ^5^, Radiation Treatment	D
39	42	F	GI	Unknown	No	Chemotherapy, Biological Treatment	L
40	63	M	Esophageal	4	No	Carboplatin, Taxol	D
41	66	M	Lung	4	Yes	Gemzar^®^ (Gemcitabine), Carboplatin	L
42	52	F	Pancreas	4	No	Gemzar^®^	D
43	62	F	Pancreas & Duodenum	4	Yes	Chemotherapy	D

^1^ F—female, M—male. ^2^ Gastrointestinal (GI) cancers gather here colorectal, colon, rectal and stomach cancers together. Patients #1 through #41 suffered from epithelial cancers, while patients #42 and #43 suffered from non-epithelial cancers, i.e., sarcomas or neuroendocrine tumors. ^3^ On 10 July 2015. L—live, D—dead. ^4^ NF1—sessile polyp in the rectum, LGD villotubular adenoma with low-grade dysplasia, waiting for surgical specimen pathology results; NF2—tubular and tubulovillous adenoma with low-grade dysplasia; NF3—large adenoma, negative surgical specimen pathology. ^5^ Acronyms: FOLFOX—a combination of the chemotherapy drugs folinic acid, fluorouracil and oxaliplatin, 5-FU—5-fluorouracil, LCV—leukocytoclastic vasculitis.

**Table 3:** Summary of the bio-ferrographic analysis and tumor markers in this study. Line background colors are aimed to easily distinguish between different disease stages and controls.

Disease	Disease Stage	No. of Valid Patients	No. of Valid Samples	No. of Invalid Patients	Sensitivity P-Phase (DOE) ^1^	Sensitivity S-Phase (DOE) ^1^	Sensitivity O-Phase (DOE) ^1^	CA-19 Detects	CEA Detects	AFP Detects
CRC and other epithelial cancers	4	21	22	4 ^2^	14% (1/7)	0% (0/4)	90% (9/10)	58% (22/38)	49% (19/39)	0% (0/4)
CRC and other epithelial cancers	3	5	8	2 ^3^	0% (0/2)	0% (0/1)	50% (1/2)	0% (0/5)	20% (1/5)	
CRC and other epithelial cancers	2	1	1	0		0/1		1/1	1/1	
CRC and other epithelial cancers	1	1	1	0	0/1					
CRC and other epithelial cancers	NF (stage 0 or unknown)	6	6	1						
Non-epithelial tumors	4	2	2	0			0/0	2/2	1/2	
Total no. of patients/samples		36	40	7						
Healthy donors—full ferrogram (negative control)	0	3	3	0	100% (3/3)					
Healthy donors—H-channel (negative control)	0		9	0						
Positive controls (C & C_R.R.)			25	0						

^1^ Calculated per patients, not per samples. Only valid (non-disqualified) samples are considered. ^2^ Five blood samples. ^3^ Three samples.

## Data Availability

The original contributions presented in this study are included in the article. Further inquiries can be directed to the corresponding author.
